# Pros and Cons of 19 Sport-Related Concussion Educational Resources in Canada: Avenues for Better Care and Prevention

**DOI:** 10.3389/fneur.2018.00872

**Published:** 2018-11-02

**Authors:** Michael D. Cusimano, Stanley Zhang, Jane Topolovec-Vranic, Ashley Grosso, Rowan Jing, Gabriela Ilie

**Affiliations:** ^1^Division of Neurosurgery, St. Michael's Hospital, Toronto, ON, Canada; ^2^Faculty of Medicine and the Dalla Lana School of Public Health, University of Toronto, Toronto, ON, Canada; ^3^Department of Occupational Science and Occupational Therapy, University of Toronto, Toronto, ON, Canada; ^4^Department of Community Health and Epidemiology, Dalhousie University, Halifax, NS, Canada

**Keywords:** concussion, mild traumatic brain injury (mTBI), concussion education and awareness programs, concussion prevention, sports injury prevention

## Abstract

**Objective:** The goal of this research was to assess the effectiveness of available concussion educational resources in Canada, the means used to disseminate this knowledge and the impact of these educational resources on players' concussion prevention knowledge.

**Methods:** We assessed concussion knowledge before and after exposure to one or more of 19 resources introduced through a national program aimed to increase awareness and knowledge of concussion. The effectiveness of the mode of delivery was measured by changes in concussion knowledge scores (CKS) between pre and pro scores.

**Measures:** Concussion knowledge scores (CKS) were calculated for pre- and post- exposure to concussion educational resources and used as a measure of both, the effectiveness of each resource as well as the effectiveness of the delivery method. The effectiveness of each educational resource was also measured by the respondents' rating of each concussion educational resource.

**Results:** Respondents in post-survey had higher CKS than those in pre-survey. Two out of the 19 newly developed concussion educational resources were effective in improving the resource users' CKS. Linear regression showed that using more resources further increased CKS. Four out of six modes of delivery enhanced respondents' concussion knowledge.

**Conclusion:** Our findings demonstrate that the newly developed Canadian concussion educational resources were effective at improving users' concussion knowledge. Our data demonstrates that using three or more resources further enhanced the users' concussion knowledge. Future research, however, is critical to assess whether concussion prevention knowledge is sufficient to reduce injuries and factors influencing it.

## Introduction

Traumatic Brain Injury (TBI) is a significant public health concern (1) and a “silent epidemic” (2) that affects approximately 1.7 million people in the United States each year from which about 275,000 are hospitalized and 52,000 die (3). In 2010 alone, it was estimated that the cost of TBIs in the United States was about $76.5 billion and $10 billion in Canada (4–6). Despite the tremendous healthcare and economic burden of TBI, a lack of knowledge in recognizing TBI or concussion is quite common and worrisome. For example, in one study, it was observed that more than half of the study's participants underestimated the prevalence of concussion and more than 30% were unaware of any return-to-play protocols (7). Other studies have identified several gaps in concussion awareness and knowledge (8, 9) including the lack of awareness of the effects of concussion on psychosocial functioning post injury, and the lack of care management post-concussion among athletes (10). However, researchers have also proposed that through educational interventions or knowledge translation activities, it may be possible to improve concussion knowledge (11–13). In a recent systematic review focusing on the effectiveness of interventions in preventing injuries in hockey (14) it was observed that rule changes could lead to a reduction in injury rate. However, only 11 studies provided conclusive evidence supporting the effectiveness of increasing concussion knowledge or awareness by educational intervention. Delivery of this knowledge was achieved through mobile app, or pamphlet (15), website (16), information sheet or seminar/presentation (17–19), physical education class (20), poster or other formats (21–25). In spite of some attempts to increase players' education, a recent study showed that over the last 20 years, old misconceptions about concussion continue to persist among school educational professionals surveyed between 1997 and 2017 (26). Although a multimodal educational intervention has been shown to positively influence primary care providers to adhere to the consensus-based guidelines in their concussion management practice (27), even after the legislation that mandated concussion education for student athletes, athletes fail to recognize concussion symptoms (28). Little is known whether or not the format of educational intervention (e.g., video vs. text) can play a role and whether or not the combination of different formats of educational interventions would be more effective in changing the behavior of non-medical professionals. Furthermore, overall little information exists to evaluate concussion educational resources with the ultimate goal to better translate concussion knowledge to the general public. To this end, the Public Health Agency of Canada (PHAC) engaged four Partners consisting of Hockey Canada (HC), Coaching Association of Canada (CAC), Canadian Centre for Ethics in Sport (CCES), and Think First (now a part of Parachute) to develop various concussion educational resources and survey thousands of Canadians about these resources. Our research group has reported the survey results and respondents' concussion knowledge previously (29, 30). The analyses we present here, however, extend previous reports by evaluating the effectiveness of the newly developed concussion resources individually to deliver concussion education, their added value to concussion knowledge, as well as evaluating the effectiveness of their different formats of delivery. We hypothesized that exposure to concussion educational resources would improve participants' knowledge and understanding of concussion.

## Methods

### Ethics

The study received approval from the Research Ethics Board of St. Michael's Hospital. Participants provided consent prior to completion of the survey.

### National surveys and concussion educational resources

This study reports on the results of two Canadian cross-sectional surveys that were administered before and after the respondents' exposure to concussion educational resources. Pre-survey responses were completed by 7,258 respondents whereas post-survey were completed by 6,031 respondents, respectively. Both surveys were described previously (29, 30). The 19 concussion educational resources evaluated are described in Table 1. A cross-sectional, anonymous, voluntary, open survey with a convenience sample of the stakeholders in the sports community in Canada, was conducted in two waves as described previously.

**Table 1:** Description of the 19 Concussion Educational Resources.

**Resource developer**	**Educational resource**	**Description**	**Pros**	**Cons**	**URL**
Hockey Canada	Concussion awareness adult mobile app (*n* = 248)	A free cell phone app outlining concussion prevention strategies, and information including definitions, official rules/ regulations in sports, facts and injury prevention and safety tips.	Free, easy to get	Relying on internet and device (cell phone access)	http://www.hockeycanada.ca/en-ca/news/2012-nr-130-en
	Concussion awareness children's mobile app (*n* = 203)	A free cell phone app including “Puckster”(bear) who educates children about injury prevention and official sport rules, definitions, management of concussions, symptoms and signs, and recovery protocol. It also featured a hockey game with Puckster.	Free, easy to get, designed for children	Relying on access to internet and device (cell phone)	http://www.hockeycanada.ca/en-ca/news/2012-nr-130-en
Coaching Association of Canada	Concussion IQ (*n* = 451)	A short quiz with eight questions, which aimed to educate users on basic signs and symptoms, prevention methods and recovery protocol. Once you select the correct answer, a detailed explanation as to why it is correct will appear.	Short and sweet, easy to do	The quiz format may not attract everyone; questions may not cover all aspects of concussion	http://www.coach.ca/what-s-your-concussion-iq–p153386
	Making headway E-learning modules (*n* = 257)	Available in six different modules aimed to educate users about prevention, concussion management, and return to play protocol. It included interactive activities including scenario videos, spot the hazardous materials in pictures and quizzes.	Nice combination of pictures, videos, and quizzes	The number of modules and scenarios are limited.	http://coach.ca/making-head-way-concussion-elearning-series-p153487
	Concussion stories video (*n* = 331)	A 5 min video of three athletes who previously had a concussion and describe their personal experiences.	Very touching stories; the video format is good for all ages.	The stories don't have all component or aspects regarding a concussion.	http://www.coach.ca/concussion-stories-p153389
	Aboriginal coaching module concussion supplement (*n* = 91)	A 30 min module delivered to rural communities across Canada. It provided written resources created to resonate with the Aboriginal population. It aimed to prevent, detect, and implement correct concussion management protocol.	A concise package with helpful information; targeted special population and audience	Delivered to limited number of qualified personnel.	http://www.coach.ca/aboriginal-coaching-modules-if-training-session-p150158
	2012 petro Canada sport leadership conference concussion session (*n* = 149)	A social event including the country's top coaches and sports scientists' joined with senior leaders from the national sport organizations to analyze, discuss, network, and learn from one other in a powerful sharing session.	An informative and effective format to connect stakeholders from various fields.	It is static; the number of participant is limited; it is more expensive than other forms of knowledge dissemination.	http://coach.ca/montreal-selected-as-host-city-for-the-2012-petro-canada-sport-leadership-sportif-conference-p141508
Canadian center for ethics in sports	Concussion 101 (*n* = 279)	A 6 min. online video (also available in a PDF format) developed by a doctor including concussion knowledge (definition, symptoms, recovery), discussing with real life examples and issues.	The video is in a cartoon format that is particular interesting for kids.	It focuses more on symptoms than management.	http://cces.ca/sites/default/files/content/docs/pdf/cces-activesafe-concussion101-e.pdf; http://cces.ca/concussion-prevention
	Active and safe pledge (*n* = 132)	A pledge formed to educate stakeholders of the importance of safety, return to play protocol, respect, awareness, responsibility and prevention of concussions.	A very useful format, especially for sport teams; it may even have long-term effects on participant.	A sense of bond between different parties is required; it is less likely to be used at individual level.	http://cces.ca/sites/default/files/content/docs/pdf/cces-activesafe-pledge-e.pdf
	Active and safe ethical decision making game (*n* = 274)	The objective is for teams to make their way from start to finish as quickly as they can. Along the way, each group will discuss and reach consensus about real-life ethical scenarios designed to encourage critical thinking about issues regarding concussion awareness, prevention and management.	Interactive and fun; Players are actively involved and decision making of each player is encourage.	Discussion around the questions for each team may vary based on their concussion knowledge.	https://cces.ca/sites/default/files/content/docs/pdf/cces-activesafe-edmgame-e.pdf
	Aboriginal concussion education and prevention module (*n* = 65)	The first of a two-part certification process, which aimed to provide culturally relevant courses for coaches and people working with Aboriginal youth. It included unique methods and perspectives not found in mainstream coaching certification programs.	Unique in coaching certification programs; specific reference to Aboriginal people	Limited audience; Some content is specific to Aboriginal people	
	Active and safe self-assessment tool for sport organizations (*n* = 177)	A scorecard for organizations to track progress in developing a concussion prevention and management program. It includes a scale from 0 (minimal) to 5 (complete), and fourteen statements targeting the topics of education, management and governance.	Nice tool to check the concussion education and management status of an organization; easy to be implemented.	It was designed specifically for sport organizations; enforcement to improve an organization's scores is critical.	https://cces.ca/sites/default/files/content/docs/pdf/cces-activesafe-selfassessment-e.pdf
	Active and safe LTAD matrix (*n* = 227)	This matrix guide outlined information regarding physical and ethical literacy, and fundamental principles based on age groups.	Age and sex are considered specifically; it is integrated into a long-term education plan.	Collaborative efforts from coaches, trainers, teachers, and even parents may be required.	https://cces.ca/sites/default/files/content/docs/pdf/cces-activesafe-ltadmatrix-e.pdf
Think first/parachute	Website (*n* = 350)	A webpage that provides current information on concussion approaches in injury prevention, and a variety of material on roles and responsibilities for all stakeholders.	Easy to access and use; can be made widely available.	The information provided is subjective to the understanding of its readers.	
	Concussion Toolkit (*n* = 200)	Included concussion management handouts for coaches, trainers, athletes, parents and medical personnel. It also included personal concussion records for each player, and an easy to follow guide for coaches and trainers.	A large amount of information tailed to specific stakeholders.	It is not interactive or dynamic. Content may need to be updated overtime.	http://www.parachutecanada.org/downloads/resources/ConcussionKit_E_2012Nov-1.pdf
	Video (*n* = 232)	A 26 min video including an interview with a neurosurgeon and professional athletes. It aimed to educate the public on concussion facts, effects on quality of life, recommended exercises, safety tips and a helmet-fitting guide.	Insight from professionals; practical knowledge and tips.	Information provided is limited.	
	Concussion education sessions (*n* = 256)	A webinar series on the brain, case studies, injury prevention strategies, signs and symptoms, myth busters, and potential short and long term effects of a concussion, and resources to assist with concussion management.	Information provided is comprehensive.	Content is more for adults than youths/kids.	http://www.parachutecanada.org/downloads/programs/activeandsafe/Overview_of_Concussion-English.pdf
	Posters (*n* = 248)	A 20“ × 28” “I am not invincible” visual poster featuring two athletes and a short summary of their personal experiences.	Touching stories; simple format for all ages.	The information provided doesn't cover all aspects of concussion.	
	Concussion cards (*n* = 359)	Two page-length cards including general information designed to aid with assessment of concussions. It included information on visible cues, signs and symptoms of a concussion, memory functions and potential red flags.	Useful and easy to make; also useful on field.	Focus on assessment and the decision to remove a player from a game may be subjective to athletes' or coaches' attitude toward concussion.	http://www.parachutecanada.org/downloads/resources/Pocket-Concussion-Recognition-Tool2013.pdf

### Study sample

As the surveys were conducted anonymously, the computer's IP address was used as a proxy for user's identification. Based on the survey data, 1,186 unique IP addresses were identified from which 503 were used in both the pre- and post-survey and 180 were used only in the pre-survey. The analysis reported here was conducted using the data set from the 503 survey respondents who completed the pre and post survey. The sample size obtained for each resource is indicated in Table 1.

### Measures

#### Demographic and social characteristics

Language, age, sex, ethnicity, education, annual household income, geographic location, and the non-medical community role the survey respondent most strongly identified with in their community were collected.

Changes in concussion knowledge scores (CKS) between pre- and post-survey testing as a measure of the effectiveness of concussion resources. As described previously (29, 30). CKS were calculated by summing the correct answers to 27 items that surveyed concussion knowledge related to physical/cognitive/mental health symptoms, concussion treatment modality, return-to-sport following a concussion, or diagnosis of concussion post head injury. The 27 items were evaluated using a dichotomous scoring process. Correct responses were given a score of one and incorrect responses were given a score of zero. Higher CKS scores indicated better knowledge of concussion. The CKS changes pre- and post- survey between the resource users and non-users as well as all respondents was used as a measure of resource effectiveness.

#### Respondents' ratings of the educational material as a measure of the effectiveness of concussion resources

Ratings were based on participants' self- reports on the level of effectiveness (not effective, neutral, or extremely effective) for each concussion educational resource.

#### Mode of delivery and its effectiveness

Each concussion resource was classified into one of six forms of delivery: (1) webpage (e.g., a website from Parachute); (2) written guidelines (e.g., active and safe self-assessment tool for sport organizations); (3) quizzes (e.g., concussion IQ); (4) videos (e.g., the concussion stories video); (5) phone apps (e.g., the children and adult's concussion awareness mobile apps); and (6) in-person resources (e.g., 2012 Petro Canada sport leadership conference concussion session). Educational mode of delivery effectiveness was evaluated by examining CKS changes between pre- and post- scores for each form of delivery.

## Data analyses

Statistical analyses were performed using SAS 9.4 4 (SAS Institute, Inc., Cary, NC, USA) and STATA 13.0 (StataCorp LLC, College Station, TX, USA). Descriptive statistics of language, sex, age, education, household income, and survey respondent location were used to describe the demographic characteristics of the survey respondents. Pearson's correlation coefficient and Wald's F test were used to compare the effectiveness of each concussion educational resource measured by changes on CKS. Student *t*-tests were performed to compare CKS between pre- and post- surveillance between the resource users and non-users in the post-survey group. An ordinary linear regression was performed with CKS as the dependent variable and age, sex, level of education, household income, use (or not use) the 19 educational resources as the independent variables. An alpha value of 0.05 or less was considered statistically significant, however, Bonferroni adjustments were applied for significance assessments to correct for experiment-wise error.

## Results

### Characteristics of survey respondents

In both pre-and post-survey, the proportion of respondents falling into each age category and the male to female ratio (2:1) remained primarily unchanged. Roughly 78% of respondents wereas between the age of 35 and 54 while less than 4% fell into the younger (< 25 years) or older respondent (>64 years) categories (*n* = 1,186, Table 2). Almost two thirds of respondents resided in Ontario, Bristish Columbia, or Alberta and reported a post secondary education at a college/university level (about 84%) with a household income greater than $20,000 (Table 2).

**Table 2:** Demographic characteristics of pre- and post- survey respondents (*n* = 1,186).

**Demographic characteristics**		**Pre-survey *n* (%)**	**Post-survey *n* (%)**
Language	English	646 (94.6)	473 (94.0)
	French	37 (5.4)	30 (6.0)
Sex	Male	420 (61.5)	331 (65.8)
	Female	261 (38.2)	170 (33.8)
	Missing	2 (0.3)	2 (0.4)
Age	15–24	24 (3.5)	18 (3.6)
	25–34	103 (15.1)	70 (13.9)
	35–44	199 (29.1)	146 (29.0)
	45–54	270 (39.5)	191 (38.0)
	55–64	73 (10.7)	66 (13.1)
	65–74	10 (1.5)	10 (2.0)
	75+	1 (0.1)	0 (0.0)
	Missing	3 (0.5)	2 (0.4)
Education level	Some high school	2 (0.3)	1 (0.2)
	High school	24 (3.5)	18 (3.6)
	Some post secondary	70 (10.2)	48 (9.5)
	Post secondary (college/university)	439 (64.3)	334 (66.4)
	Masters degree	80 (11.7)	56 (11.1)
	Doctoral degree	7 (1.0)	5 (1.0)
	Professional designation	47 (6.9)	32 (6.4)
	Other	8 (1.2)	7 (1.4)
	Missing	6 (0.9)	2 (0.4)
Household income	$0–$20,000	6 (0.9)	7 (1.4)
	$20,001–100,000	271 (39.7)	185 (36.8)
	$100,001+	274 (40.1)	204 (40.6)
	Missing	132 (19.3)	107 (21.2)
Location	Ontario	249 (36.5)	179 (35.6)
	Alberta	109 (16.0)	77 (15.3)
	British Columbia	86 (12.6)	72 (14.3)
	Manitoba	74 (10.8)	52 (10.3)
	Saskatchewan	49 (7.2)	37 (7.4)
	Quebec	43 (6.3)	38 (7.6)
	New Brunswick	29 (4.2)	18 (3.6)
	Nova Scotia	20 (2.9)	11 (2.2)
	Newfoundland and Labrador	10 (1.5)	8 (1.6)
	Prince Edward Island	7 (1.0)	3 (0.6)
	Yukon	6 (0.9)	7 (1.4)
	Outside Canada	1 (0.1)	1 (0.1)

*CAD dollars - representing house hold annual income*.

### Effectiveness of concussion resources measured by CKS change

Group comparisons were performed and results showed that both resource users and non-users in the post-survey had significantly higher CKS than at pre-survey while users' CKS were significantly higher than that of non-users (Table 3).

**Table 3:** Comparison of CKS between respondents of the pre- and post- survey as well as between users and non-users in post-survey.

**Pre-post analysis**	***n***	**Mean ± SD**
Pre-survey	683	20.1 ± 3.7
Post-survey Non- users	326	22.3 ± 3.3^*^
Post-survey users	177	23.1 ± 3.2^*^

* *F_(1, 1185)_ = 72.38, p < 0.0001. Post-hoc tests showed that all the pair-wise comparisons were significant*.

Linear regression analyses showed that level of education affected the respondents' CKS. A higher level of education was associated with higher CKS overall. However, age, sex, and income were not statistically significantly associated with changes in the respondents' CKSs from pre to post test. Results also indicated that the use of Concussion IQ and Concussion Cards was the only form of concusion educational resource that statistically significantly increased respondents' CKS from pre to post survey (Table 4).

**Table 4:** Ordinary linear regression analysis of the prediction of CKS based on individual concussion resource use and demographics.

**Independent variable**	**Coefficient estimates (SE)**	**95% Confidence interval**
Sex	−0.05 (0.22)	(−0.48, 0.38)
Age	0.004 (0.10)	(−0.19, 0.20)
Household income	0.09 (0.062)	(−0.03, 0.21)
Highest education	0.25^***^ (0.09)	(0.07, 0.42)
HC adult app use	1.78 (0.96)	(−0.12, 3.68)
HC children app use	−0.97 (1.26)	(−3.44, 1.50)
CAC concussion IQ use	1.31^**^ (0.58)	(0.16, 2.46)
CAC E–learning use	1.11 (0.89)	(−0.64, 2.86)
CAC concussion stories video use	−0.38 (0.82)	(−2.00, 1.24)
CAC aboriginal learning module use	0.68 (1.43)	(−2.13, 3.48)
CAC petro leadership Conference Use	1.08 (1.07)	(−1.02, 3.18)
CCES concussion 101 use	0.71 (0.87)	(−1.00, 2,42)
CCES active and safe pledge use	−1.71 (1.30)	(−4.25, 0.84)
CCES active and safe decision–making game use	0.88 (0.78)	(−0.64, 2.40)
CCES Aboriginal concussion education module use	−4.17 (2.13)	(−8.36, 0.10)
CCES active and safe self–assessment use	−1.39 (1.27)	(−3.88, 1.11)
CCES LTAD matrix use	−0.74 (1.06)	(−2.84, 1.34)
TF website use	0.19 (0.85)	(−1.48, 1.86)
TF concussion web toolkit use	1.19 (0.98)	(−0.72, 3.11)
TF video use	0.33 (0.96)	(−1.56, 2.22)
TF concussion education session use	1.58 (0.84)	(−0.07, 3.24)
TF poster use	1.49 (0.99)	(−0.46, 3.44)
TF card use	1.88^***^ (0.64)	(0.62, 3.14)
Constant	19.37^***^ (0.71)	(17.97, 20.77)

** *indicates significance at 5% level*;

*** *indicates significance at 1% level*.

*HC, Hockey Canada; CAC, Coaching Association of Canada; CCES, Canadian Center for Ethics in Sport; TF, Think First (now Parachute)*.

**Figure 1:**
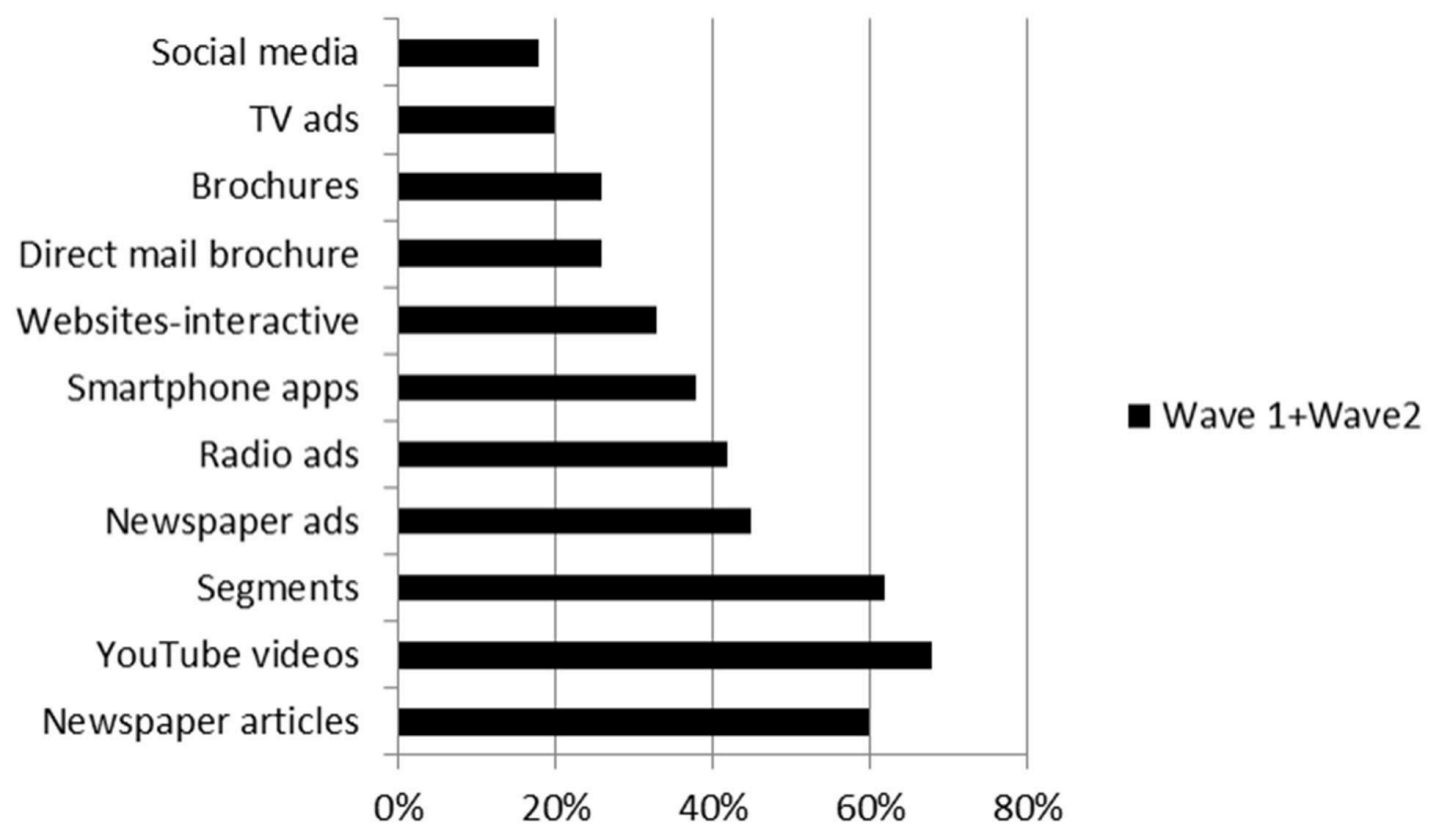
(1.1). Means and standard errors for CKS for the distribution of number of concussion educational resources used by respondents. Figure (1.2). Percentage of concussion educational resources used by respondents.

Importantly, using three or more resources was predictive of a statistically significant increase in CKS than using a single resource (Figure 1).

### Effectiveness of concussion resources related to mode of delivery

**Figure 2:**
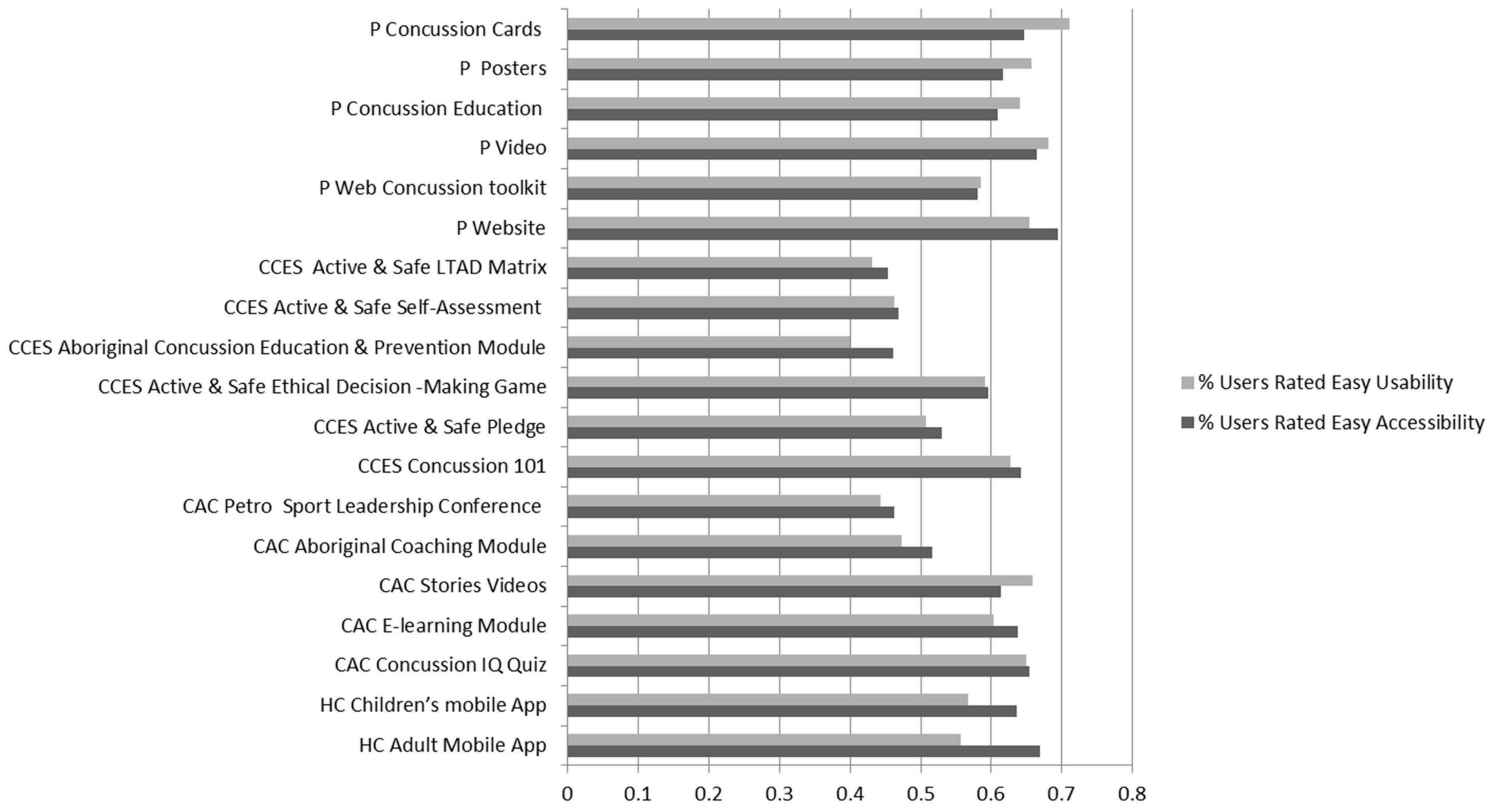
(2.1) Means and standard errors for CKS pre-, post-survey users, and post-survey non-users stratified by mode of delivery. CKS means for both users and non-users post-survey were statistically significantly higher (*p* < 0.0001) than pre-survey CKS means. ^*^p < 0.05, ^**^p < 0.001. (2. 2) Proportion of concussion educational resources rated by users in terms of ease of usability and accessibility.

The mode of delivery for all concussion educational resources was analyzed to determine the most effective method of distribution of concussion knowledge and awareness resources. Based on the mode of delivery, CKS of respondents who used any of the concussion resources in post-survey were compared to that of those who were not exposed to such resources in pre-survey (Figure 2) or those who did not use such resources in post-survey (Figure 2).

When comparing resource users with non-users in the post- survey, we found that 4 out of 6 modes of delivery significantly increased the users' CKS. Quizzes and videos were the only two delivery methods that failed to impact CKS (Figure 2). When compared, there was no significant difference found among different educational format modalities (*p* > 0.05).

### Effectiveness of concussion resources measured by respondents' rating

**Figure 3:**
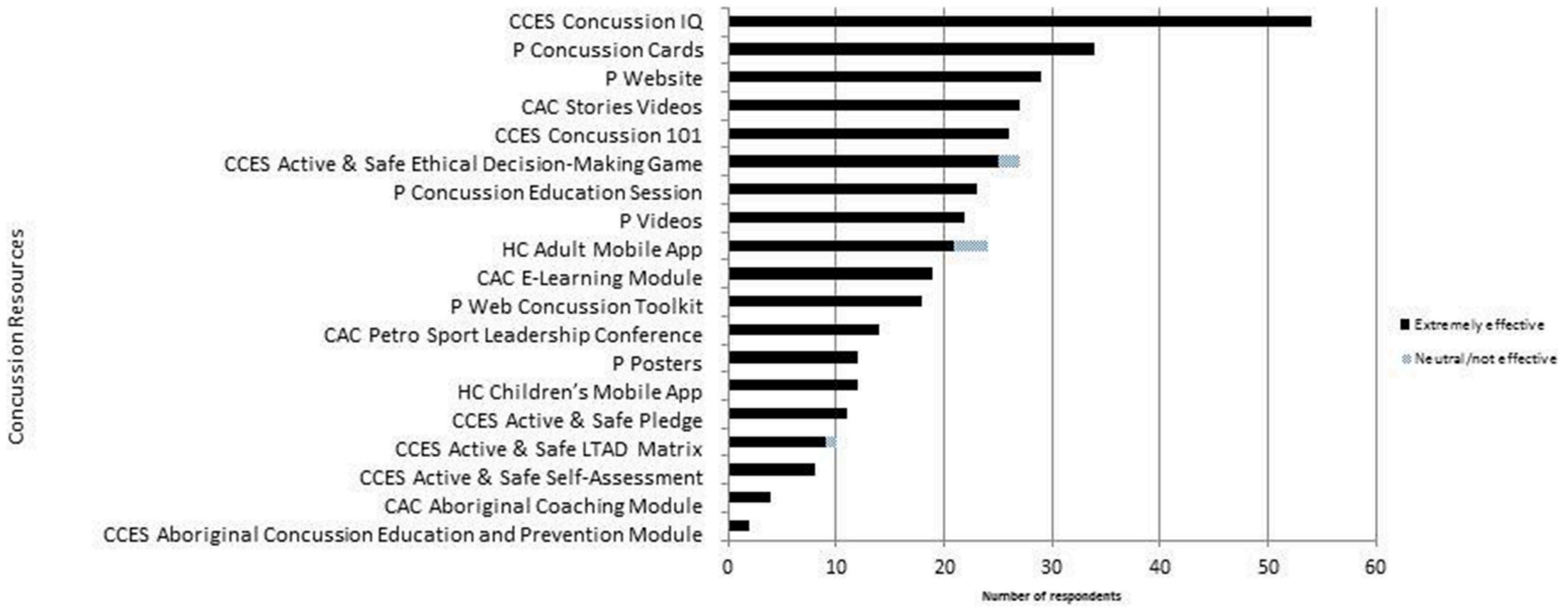
Number of respondents and their perceived effectiveness of the concussion resources evaluated.

Respondents were asked to rate the effectiveness of each concussion resource (Figure 3). Overall, most resources were rated as “extremely effective” (Figure 3). However, a regression analysis on the correlation between respondents' ratings and their CKS did not reveal a statistically significant associations (*p* > 0.05).

## Discussion

### Main findings

The results presented illustrate that educational resources delivered at a national level to athletic communities can improve knowledge of concussion. We found that 2 out of 19 resources and 4 out of 6 modes of concussion education delivery were successful in achieving this goal. The webpage educational resource was the most effective mode of delivery concussion knowledge. In addition, our results showed that those who used three or more educational resources had more significant improvements in their concussion knowledge.

#### Implications

Studies in concussion education have tried various approaches to promote autonomy in online learners (31–33) from written guidelines for athletes, coaches, educators and parents, brochures, quizzes, videos, and websites (34) to tailoring educational interventions to specific needs, skills, preferences, and the culture of the consumer in order to enhance the effectiveness of such educational resources (35). Indeed, autonomous self-regulated learning is emphasized in Piaget's constructivist learning and teaching theory (36) and has been identified as one of the most effective ways to promote behavioral change and active learning, long term (37–39). Yet much remains to be done to change engrained cultural perceptions of concussions and their effects on the individuals, their family and community teaching over time. It takes a community to make changes such as these and stakeholders at all levels that exert an influence on behavior, from the individual, to sport teams, schools, parents, coaches, teams, cities, provinces, and the government of the country in which players reside. Together these bodies of influence could impact greater change and more rapidly than any one level of influence operating alone. Governments through the ministries of education and health, could exert influence through legislation, could collaborate with physician groups to improve levels of education among physicians and other health professionals, school teachers athletic lead trainers. Parents could facilitate collaborations and knowledge translation by becoming engaged at multiple levels of athlete education—schools, clubs, leagues, and provincial and national sports organizations.

The present study supports the need to tailor concussion educational resources for the intended audience but many questions remain unanswered and could be addressed with a larger sample size. For the studied populations, age did not affect their CKS, regardless of the educational resources or mode of delivery used. This may not be the case if very young athletes (e.g., 15 years old or younger) are the targeted population as their knowledge base and reading comprehension are quite different (16). Similarly, income was not a significant predicting factor of CKS for the participants in this study as the group with very low income (< $20,000) was very small (0.9%).

The finding that respondents were able to improve their concussion knowledge in a short period of time suggests these participants may have been motivated learners. According to the health belief model, they may be motivated by (1) the desire to avoid illness, or get well, if they already have had a concussion, and (2) the belief that knowing more about concussion will help prevent or cure it (40). With the learning paradigm shifted from 3 R's (reading, ‘riting, ‘rithmetic) to 4 Es (exposing knowledge, employing information, expressing ideas compellingly, and ethics-right and wrong on the information highway) in the digital era (41), online concussion educational resources are fashioned with several benefits pertaining to eHealth such as avoiding unnecessary delays in receiving the resources and getting advice/new knowledge from authorities (e.g., concussion experts' opinion and up-to-date concussion guidelines) (42) and reaching a large audience (43). Unfortunately, the knowledge gained from such educational interventions may not last for a long time (44), suggesting that such educational efforts should be made on a regular basis or ongoing, and based on our results, combining different formats of delivery can achieve more knowledge gains. Nevertheless, it is important to note that the effectiveness of concussion educational resources doesn't necessarily translate to attitude or behavioral changes (especially in coaches, results not shown), which has been reported in several studies (45–47).

### Limitations

Although we had a large sample size, our desire to do a paired analysis restricted that sample size to just over 1,100 responses before and after educational resource exposure. Readers must be cautions interpreting these results as using a voluntary sampling method may exclude certain demographic or ethical groups and using IP addresses as a proxy for the same respondent may overestimate the number of unique respondents. While the results may not be representative of the entire country, Ontario, British Columbia, and Alberta constitute over half of the Canadian population rendering our results some breadth. Since the distribution of concussion education resources was disseminated via online and mobile applications, the targeted audience was limited by the availability and accessibility of survey respondents to the Internet. Another limitation is the possibility that a respondent's improved knowledge regarding concussion education happened on their own accord and did not stem from the use of the concussion resources we presented. This may have posed a maturational threat of internal validity and potentially impacted the outcome of the study. Further, data were not collected for other events that might have influenced levels of knowledge and awareness. Lastly our results could have been influenced by events such as media reporting of concussion or other education initiatives made available throughout the time period of this survey.

## Conclusions

Despite these limitations the results presented here have merit and warrant follow up investigations. Our findings demonstrated that the concussion educational resources we provided, based on respondents' results, were able to improve user concussion knowledge. Using three or more educational resources appeared to be more effective than single resources. Future research is needed to evaluate whether implementing these concussion educational resources translate into fewer injuries short and long term.

## Ethics statement

This study was carried out in accordance with the recommendations of Tri-Council Policy Statement guidelines, Research Ethics Board at St. Michael's Hospital with implied informed consent from all subjects. All subjects gave informed consent in accordance with the Declaration of Helsinki. The protocol was approved by the Research Ethics Board at St. Michael's Hospital.

## Author contributions

MC was responsible for conception and design as well as analysis and interpretation of the data and revising the manuscript. JT-V, SZ, RJ, AG and GI contributed to the analysis and interpretation of the data as well as revising of the manuscript.

### Conflict of interest statement

The authors declare that the research was conducted in the absence of any commercial or financial relationships that could be construed as a potential conflict of interest.
